# Pattern of Abuse in Children With Behavioral Disorders Presenting to a Tertiary Care Hospital in Peshawar

**DOI:** 10.7759/cureus.20379

**Published:** 2021-12-13

**Authors:** Amir Muhammad, Khawaja Kamran Wajid, Bibi Alia, Rabiya Munir, Muneeba Jan Bahadur, Uswa Matloob

**Affiliations:** 1 Pediatrics, Lady Reading Hospital, Medical Teaching Institution (MTI), Peshawar, PAK; 2 Pediatrics and Neonatology, Lady Reading Hospital, Medical Teaching Institution (MTI), Peshawar, PAK; 3 Pediatric Medicine, Lady Reading Hospital, Medical Teaching Institution (MTI), Peshawar, PAK; 4 Psychiatry and Behavioral Sciences, Lady Reading Hospital, Medical Teaching Institution (MTI), Peshawar, PAK

**Keywords:** physical abuse, children, sexual abuse, behavioral disorder, child abuse

## Abstract

Objective

To determine the different types of child abuse and its association with behavioral disorders in children presenting to a tertiary care hospital.

Materials and methods

One hundred abused Pakistani children, of both genders, were included. Children with cerebral palsy, a neurodegenerative disorder, chronic illness, chronic liver disease, congenital heart disease, chronic renal failure, and parents who refused to become part of the study were excluded. Descriptive statistics were calculated. The chi-square test was applied to compare the pattern of abuse among various types of behavioral disorders. P≤0.05 was considered significant.

Results

The mean age of the study was 10.38±2.64 years. The total number of males was 51 (51%); the rest (49; 49%) were females. The most common behavioral disorder was functional disorder (n=59, 59%) followed by depression (n=26, 26%). The most frequent abuse was physical (n=87, 87%) followed by verbal (n=7, 7%) and the least was sexual (n=6, 6%). There was no statistically significant association between type of behavioral disorder and type of abuse in children (P=0.162).

Conclusion

The most common type of child abuse among children with behavioral disorders is physical abuse followed by verbal. The type of behavioral disorder is not associated with a specific type of child abuse.

## Introduction

The four most common types of child abuse are physical abuse, sexual abuse, emotional abuse, and neglect [1]. Around the world, in one year, about one billion children are affected by emotional, physical, or sexual abuse [2]. Children in developing countries are abused by parents, family members, health workers, employers, and teachers [3]. Sexual abuse, in most cases, is extrafamilial [4]. It is shown that non-schoolgoing children become victims of abuse more often [4]. The female gender is more prone to psychological abuse than males while males are more victims of physical abuse [5]. Many factors play a role in child abuse; the most common of them are childhood experiences of abuse, parental educational level, socioeconomic level, violence at home, gender, and age [6-8]. In our country, according to an unofficial report, about 20% of children suffer from sexual abuse [3]. Behavioral disorders are frequently found in children less than 10 years of age. These include functional disorders, attention deficit hyperactivity disorder (ADHD), depression, and stress [9]. In functional disorders, abnormal movements of body parts in the absence of physical injury or disease are seen. In ADHD, the child is unable to sit calmly for a certain time, has a lack of compliance, and shows a lack of attention [10]. Studies conducted on the Brazilian population and Japan have been shown a statistically significant relationship between child abuse and these disorders [11-12]. There is a lack of proper reporting on child abuse in our country. Children with behavioral disorders are more prone to abuse. This is the first study of its kind on the abuse of children with behavioral disorders. Child abuse can result in various disastrous consequences like quitting school, inability to take interest in studies, and failure to become a responsible citizen. The objective of this study was to determine different types of child abuse and their association with behavioral disorders in children presenting to a tertiary care hospital.

## Materials and methods

This prospective study was conducted at the department of pediatrics of a tertiary care hospital - Medical Teaching Institution (MTI), Lady Reading Hospital (LRH), Peshawar - from January 2019 to December 2020. A total of 100 cases were included in the study by an arbitrary method. Ethical approval was obtained from the hospital ethics committee. Verbal and written consent was obtained from parents of children after a detailed explanation. All children with behavioral disorders and who were physically abused were included. Children with cerebral palsy, a neurodegenerative disorder, chronic illness, chronic liver disease, congenital heart disease, chronic renal failure, and parents who refused to become part of the study were excluded. A detailed history and medical examination of all participants were done by a psychologist and pediatric consultant. Age, gender, the pattern of abuse (physical, verbal, or sexual), type of behavioral disorders (functional disorder, ADHD, depression, or stress), child schooling, parental socioeconomic status, and paternal addiction were recorded. Behavioral disorders were diagnosed clinically by a consultant psychiatrist. The Statistical Package for the Social Sciences (SPSS), ver 22 (IBM Corp., Armonk, NY) was used for data analysis. Quantitative variables like age were expressed as mean and standard deviation. Qualitative variables like gender, pattern of abuse, and type of behavioral disorder were expressed as frequency and percentages. They were considered significant.

## Results

The mean age of the participants was 10.38±2.64 years, ranging from four to 10 years. Most parents of abused children had a lower socioeconomic status (n=63, 63%) followed by lower-middle (n=2, 25%). Parental addiction was found in 68% of cases. Only 3% of parents were government employees. All the participants were studying in school (Table 1). The most common type of behavioral disorder was functional disorder (n=59, 59%) followed by depression (n=26, 26%). The most frequent type of abuse was physical (n=87, 87%) followed by verbal (n=7, 7%) and the least frequent was sexual (n=6, 6%) (Table 2). There was no statistically significant association between the type of behavioral disorder and the type of abuse in children (P=0.162). The detailed statistics are given in Table 3.

**Table 1:** Demographics of the participants

		Frequency	Percent
Gender of the participant	Male	51	51.0
	Female	49	49.0
Socioeconomic status of parent	Lower	63	63.0
	Lower middle	25	25.0
	Upper middle	12	12.0
Paternal addiction	Yes	68	68.0
	No	32	32.0
Parental employment	Self-employed	97	97.0
	Govt servant	3	3.0
Child's schooling	Yes	100	100.0

**Table 2:** Pattern of behavioral disorder and child abuse ADHD: attention deficit hyperactivity disorder

		Frequency	Percent
Behavioral disorder	Functional disorder	59	59.0
	ADHD	8	8.0
	Depression	26	26.0
	Stress	7	7.0
Type of abuse	Physical	87	87.0
	Sexual	6	6.0
	Verbal	7	7.0

**Table 3:** Association of type of behavioral disorder and type of abuse in children *Fisher exact test. ADHD: attention deficit hyperactivity disorder.

Behavioral disorder	Type of abuse						P-value^*^
	Physical		Sexual		Verbal		
	N	%	n	%	n	%	
Functional disorder	53	60.9	2	33.3	4	57.1	0.162
ADHD	7	8.0	0	0.0	1	14.3	
Depression	23	26.4	2	33.3	1	14.3	
Stress	4	4.6	2	33.3	1	14.3	

## Discussion

This study was conducted to determine various types of abuse in children with behavioral disorders. Our finding showed that the most common behavioral disorder was functional disorder and depression. The most frequent abuse was physical and verbal, and the least was sexual.

Our results revealed that the most common disorder in our study population was functional disorder. In this type of disorder, abnormal movement of body parts, such as tremors or weakness, occurs without any injury or disease. A previous study also reported that the most common type of disorder was functional [13].

The term physical abuse is used for a non-incidental injury caused by parents or other persons. Physical abuse can result in cuts, red marks, welts, bruising, muscle sprains, or fractured bones. Some social cultures also come under child abuse like inserting sharp objects for healing wounds, etc. [14]. Our study showed that the most common sort of child abuse was physical. Previous studies also showed that the most common type of abuse among children with behavioral disorders was physical [13-15].

Verbal abuse was the second most common type of child abuse in our study. Examples of verbal abuse are hostility, constant rejection, bullying, teasing, criticism, yelling, and exposure to family violence. The consequences of verbal abuse are similar to that of physical abuse [12]. Moura et al. conducted a study in Brazil on the prevalence of bullying victims among school-age children with behavioral disorders. Their results showed that the most frequent types of child abuse were verbal, followed by physical [11]. These results are a little different from the pattern of child abuse in our study. The difference can be attributed to the social and educational levels of parents and other people coming in contact with children with behavioral disorders.

Most of the abused children in our study were from low socioeconomic levels. This can be due to the fact that poor parents cannot give adequate time to safeguard their children and, as a result, these children are more prone to abuse and victimization. Zielinski et al. conducted a study on the relationship between child abuse and socioeconomic level, and their findings revealed that children from a low socioeconomic background are more prone to child abuse [16].

Our research showed no statistical association between various types of behavioral disorders with the type of child abuse. These results showed that children with any sort of behavioral disorder can suffer from abuse. To our knowledge, no such comparison had been reported in the literature.

## Conclusions

Within the limits of this research, it can be concluded that the most common types of child abuse among children with behavioral disorders are physical followed by verbal. The type of behavioral disorder is not associated with a specific type of child abuse.
